# A mini‐review of chronic suppression of the hypothalamic–pituitary–adrenal axis in animals with P‐glycoprotein deficiency

**DOI:** 10.1111/jsap.70098

**Published:** 2026-02-03

**Authors:** K. L. Mealey

**Affiliations:** ^1^ Program in Individualized Medicine (PrIMe), Department of Veterinary Clinical Sciences College of Veterinary Medicine Washington State University Pullman Washington USA

## Abstract

This mini‐review describes the pathophysiology of the disrupted hypothalamic–pituitary–adrenal axis in dogs, and probably cats, with the MDR1 (ABCB1) mutation, ABCB1‐1Δ in dogs and *ABCB1*1930‐1931del TC in cats. Clinical consequences in affected animals are discussed and therapeutic interventions to consider are presented.

## INTRODUCTION

P‐glycoprotein, the multidrug efflux transporter, is most well known in veterinary medicine as a component of the blood–brain barrier that prevents potentially toxic drugs from gaining access to the central nervous system and as a component of biliary canaliculi where it enhances biliary drug excretion (Mealey et al., 2023). Specifically, P‐glycoprotein is expressed on the luminal border of brain capillary endothelial cells. When P‐glycoprotein substrates (*i.e*. ivermectin, loperamide and many other drugs) diffuse into these endothelial cells, P‐glycoprotein pumps them back into the capillary lumen, curbing their entry into the central nervous system (CNS). Regarding its role in biliary excretion, P‐gp is expressed on the biliary canalicular border of hepatocytes where it pumps substrates into bile (Geyer & Janko, 2012).

Less well known is P‐glycoprotein's involvement in the hypothalamic–pituitary–adrenal (HPA) axis. Cortisol, the key hormone involved in HPA axis feedback inhibition, is a substrate for P‐glycoprotein (Uhr et al., 2002), which means that the concentration of cortisol reaching the CNS is restricted by P‐glycoprotein at the blood–brain barrier. As a result, mice with P‐glycoprotein deficiency, and likely dogs and cats with the MDR1 mutation, accumulate higher concentrations of cortisol within the CNS, exerting greater feedback inhibition on the hypothalamus (Müller et al., 2003). Affected animals have abnormal adrenal function tests and experience clinical signs consistent with glucocorticoid deficiency (*e.g*. eunatraemic, eukalaemic hypoadrenocorticism) (Mealey et al., 2006; Müller et al., 2003). Most research involving P‐glycoprotein's HPA axis involvement has been performed in rodents and will be summarised below. Two published studies compared components of the HPA axis of normal dogs relative to that of dogs with the MDR1 mutation. The author has two goals in writing this review: (i) to raise awareness of this issue among clinical veterinarians in order to improve patient outcomes and (ii) stimulate further investigation into this phenomenon in P‐glycoprotein‐deficient dogs and cats.

## THE HPA AXIS

**FIG 1:**
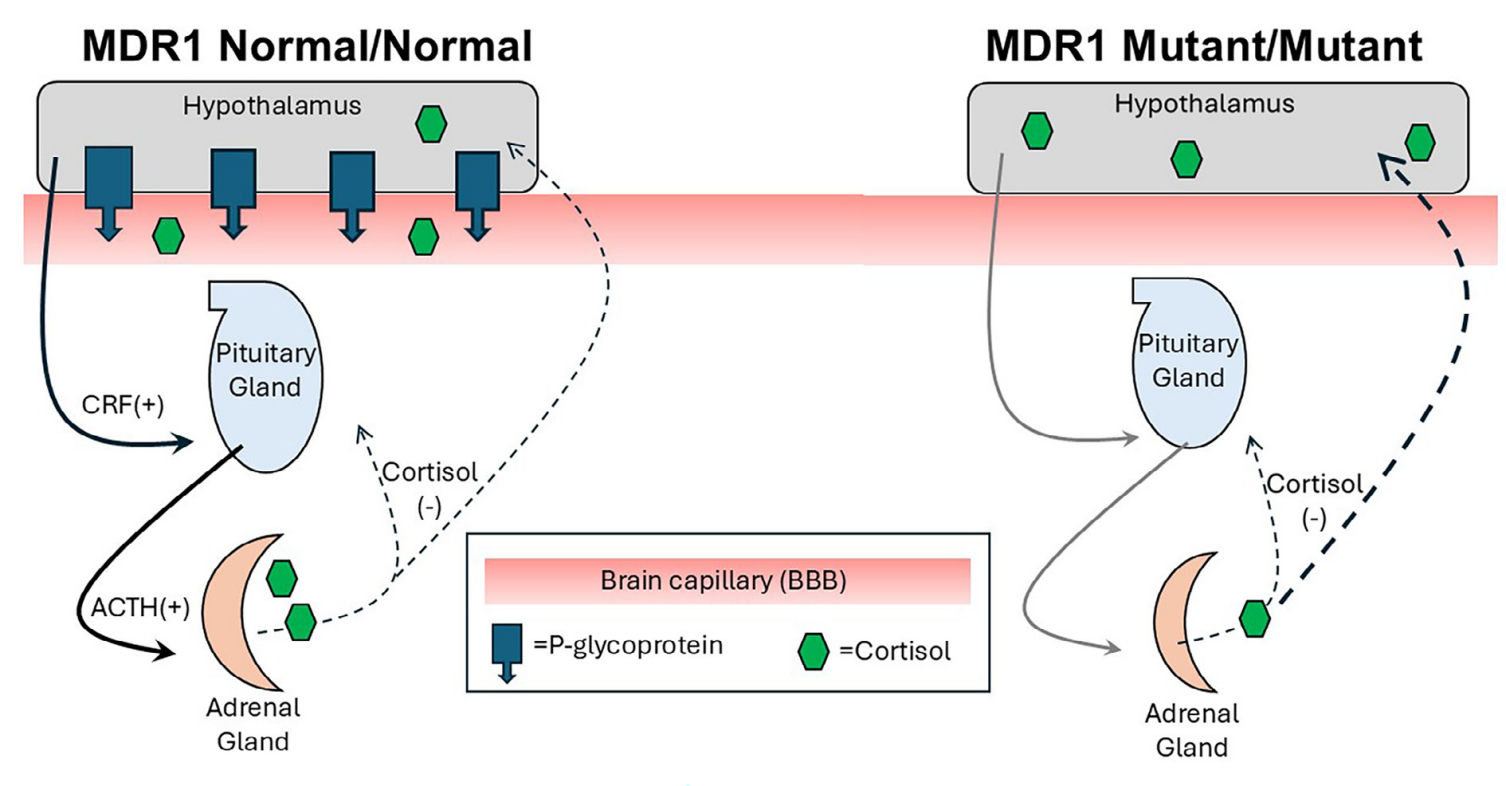
Schematic of the tissues and hormones involved in HPA axis function in both P‐glycoprotein‐replete (left) and ‐deficient (right) states, including the respective stimulatory or inhibitory hormonal effects.

Fig 1 diagrams the tissues and hormones involved in HPA axis function in both P‐glycoprotein‐replete and ‐deficient states, including the respective stimulatory or inhibitory hormonal effects. An animal's ability to adapt to stress requires activation of the HPA axis when glucocorticoids are needed for mobilising energy reserves to respond to real or perceived homeostatic challenges. Equally important is de‐activation of the HPA axis because there is a substantial energy cost to the animal under conditions of unnecessary or prolonged HPA axis activation. When an animal perceives or experiences a stressor insult, distinct neurons in the hypothalamus release corticotropin‐releasing hormone (CRH). These neurons release CRH into the hypophyseal portal veins which deliver CRH to corticotropic receptors in the anterior pituitary gland. Binding of CRH to these receptors stimulates second messenger systems that eventually cause adrenocorticotropic hormone (ACTH) to be secreted into the systemic circulation (Herman et al., 2016). Upon reaching the adrenal cortex, ACTH binds to melanocortin receptors on the surface of zona fasciculata cells with the downstream effect of increasing glucocorticoid (cortisol) secretion into the circulation. While in circulation, cortisol exerts its metabolic effects but is also available to bind glucocorticoid receptors in the anterior pituitary gland and the paraventricular hypothalamic nucleus, repressing transcription of genes responsible for ACTH and CRH synthesis, respectively. The ability of cortisol to reach CNS glucocorticoid receptors is where P‐glycoprotein influences the HPA axis.

## P‐GLYCOPROTEIN'S CONTRIBUTION TO THE HPA AXIS

Because cortisol is a substrate for P‐glycoprotein (Uhr et al., 2002), its concentration within the CNS, where it can bind hypothalamic glucocorticoid receptors, is highly restricted relative to its concentration in the systemic circulation. Limiting cortisol's access to the hypothalamus thereby limits its ability to suppress CRH release (Fig 1). By comparison, P‐glycoprotein‐deficient animals are unable to restrict cortisol's access to the CNS (Müller et al., 2003). Consequently, more cortisol is available to bind hypothalamic and anterior pituitary glucocorticoid receptors, resulting in pronounced suppression of CRH and ACTH release. To summarise, the hypothalamus and anterior pituitary are exposed to greater feedback inhibition by cortisol in animals with P‐glycoprotein deficiency than in “normal” animals, curbing CRH and ACTH release. Depressed synthesis of CRH and ACTH results in lower levels of cortisol synthesis and secretion by the adrenal glands of P‐glycoprotein‐deficient animals compared to those with normal P‐glycoprotein function under equivalent homeostatic conditions. It is important to note that because aldosterone production is not inhibited by cortisol feedback, electrolyte abnormalities associated with mineralocorticoid deficiency are not a component of HPA axis suppression in P‐glycoprotein‐deficient animals.

## EVIDENCE OF HPA AXIS ABERRATIONS IN ANIMALS WITH P‐GLYCOPROTEIN DEFICIENCY

The first report implicating P‐glycoprotein as a component of the HPA axis was published in 2002 (Uhr et al., 2002). The investigators demonstrated that *mdr1ab*(−/−) mice (genetically engineered P‐glycoprotein knockouts) accumulated twofold higher concentrations of corticosterone and 4.5‐fold higher concentrations of cortisol in brain tissue than their wildtype counterparts. The investigators postulated that P‐glycoprotein‐mediated restriction of cortisol and corticosterone access to the hypothalamus profoundly influences the HPA system under conditions of normal homeostasis and stress. The same research group (Müller et al., 2003) followed up with functional studies of the HPA axis in *mdr1ab*(−/−) mice compared to wildtype mice. Their results indicated that, relative to wildtype mice, *mdr1ab*(−/−) mice had consistently lower plasma ACTH concentrations, lower corticosterone plasma concentrations at the circadian peak and after stress, lower CRH messenger RNA expression in the hypothalamus and required lower doses of dexamethasone to suppress corticosteroid plasma concentrations. The investigators concluded that *mdr1ab*(−/−) mice experienced sustained suppression of the HPA axis, likely impeding their ability to cope with homeostatic disruption. They postulated that P‐glycoprotein deficiency in humans could enhance susceptibility to stress‐associated psychiatric disorders (Müller et al., 2004).

The author identified one report in which HPA axis function in dogs with P‐glycoprotein deficiency (homozygous for the MDR1 mutation) was compared to that of wildtype dogs and another study that compared the urinary cortisol metabolome in P‐glycoprotein‐deficient and wildtype dogs. The first study (Mealey et al., 2006) was comprised of both MDR1 mutant/mutant (*n* = 4) and wildtype (*n* = 3) dogs and yielded statistically significant differences despite the limited number of dogs in each group. The former group (P‐glycoprotein deficient) had significantly lower concentrations of basal plasma cortisol, ACTH‐stimulated plasma cortisol and ACTH plasma concentrations after dexamethasone administration. The second study (Gramer et al., 2022) employed metabolomics to compare urine concentrations of cortisol and 13 cortisol metabolites in samples collected from 23 MDR1 mutant/mutant (*n* = 23; 15 Collies and eight Australian shepherds) and wildtype (*n* = 16; nine Collies and seven Australian shepherds) dogs. Results indicated that concentrations of all cortisol metabolites were lower in urine of MDR1 mutant/mutant dogs than wildtype dogs, with allo‐tetrahydrocortisol and β‐cortol achieving statistical significance. Collectively, results of both studies provide evidence that the HPA axis in P‐glycoprotein‐deficient dogs is chronically suppressed relative to normal dogs. It is reasonable to infer that these dogs, and potentially P‐glycoprotein‐deficient cats, are unable to mount an appropriate stress response during states of altered homeostasis (“stress”) such as severe illness, extreme physical exertion or emotional stress such as that caused by separation anxiety or loud noises. Numerous observations by veterinarians and pet owners as well as diagnostic testing results in affected animals lend credence to this inference and will be described below.

Currently, there are no studies reporting on the HPA axis of cats with P‐glycoprotein deficiency. A case report describes use of intravenous hydrocortisone in a critically ill Maine Coon cat suspected of having critical illness‐related corticosteroid insufficiency (CIRCI) based on persistent hypotension unresponsive to fluid and vasopressor therapy (Pisano et al., 2017). Within 3 hours, normotension was achieved and maintained. The cat's MDR1 genotype was not determined, but it is worth noting that the Maine Coon is the breed with the highest frequency of the feline MDR1 mutation. The cat had received megestrol acetate 2 months prior to this hypotensive incident, and while megestrol acetate can suppress the HPA axis, the duration of this suppression persists for up to 2 to 4 weeks, not 2 months (Church et al., 1994; Watson et al., 1989). Whether this cat had chronic HPA axis suppression due to P‐glycoprotein deficiency is unknown, particularly since ACTH stimulation was not performed, but it is reasonable to assume that cats with the MDR1 mutation, like dogs and mice, would have a blunted HPA axis and exhibit the clinical signs of CIRCI.

## CLINICAL CONSEQUENCES OF HPA AXIS SUPPRESSION IN ANIMALS WITH P‐GLYCOPROTEIN DEFICIENCY

After it was determined that *abcb1ab*(−/−) mice experience chronic HPA axis suppression, studies were undertaken to determine if this would adversely affect behavioural responses to stress (Schoenfelder et al., 2012). A group of abcb1*ab*(−/−) mice was compared to a group of wildtype mice after both groups were subjected to a series of behavioural assessments including the forced swimming test, open field test, elevated plus maze test and tail suspension test. Significant phenotypical differences were observed between the groups indicating an impaired stress response in *abcb1ab*(−/−) mice compared to wildtype mice. Administration of exogenous corticosterone improved performance in *abcb1ab*(−/−) mice but not in wildtype mice. The investigators concluded that mice lacking P‐glycoprotein are adversely impacted under conditions of stress compared to mice with normal P‐glycoprotein function (Schoenfelder et al., 2012).

Table 1 provides comments from veterinarians about the nature/clinical behaviour of severely ill Collies, the breed with the highest frequency of the MDR1 mutation (75%) (Neff et al., 2004). These comments, which were written prior to any publications on the role of canine P‐glycoprotein in the HPA axis, describe exactly what one would expect in a patient unable to mount an adequate stress response. The Collies these veterinarians treated may have been experiencing glucocorticoid deficiency akin to what has been proposed to be relative adrenal insufficiency or CIRCI (Martin, 2013). Proposed clinical signs of CIRCI include depression, weakness, gastrointestinal abnormalities and hypotension refractory to fluid resuscitation (Martin, 2013). There is not a true definition of CIRCI in veterinary medicine so it is difficult to determine if the suppressed HPA axis in P‐glycoprotein‐deficient animals might be a subcategory of a CIRCI‐like phenomenon or a separate entity. However, both seem to respond to low‐dose corticosteroid supplementation. Additional discussion about potential treatment options for P‐glycoprotein‐deficient patients will be discussed in the next section.

**Table 1:** Comments made by veterinarians about severely ill Collies on an internet veterinary discussion forum^†^

Collies do not participate in their own recovery Collies are “wimpy” Collies have no will to live Collies just lay down and die

^†^ Veterinary Information Network

In the decade since the blunted HPA axis in dogs homozygous for the MDR1 mutation was reported, many veterinarians, pet owners and breed clubs have contacted the author to discuss their patient, pet or breed. In some instances, medical records were shared. The remainder of this section will summarise these discussions, representing several dozen individual cases, which fall into three general categories (Table 2). The first general category includes dogs of known genotype (MDR1 mutant/mutant or MDR1 mutant/normal) with a diagnosed medical condition (*i.e*. pancreatitis, pyometra, pneumonia, trauma) that not only fails to respond well to traditional treatments but the dogs appear much sicker than biochemical and/or imaging parameters would indicate. Veterinarians often describe small adrenal glands on abdominal ultrasound, low basal cortisol and an unimpressive post‐ACTH cortisol plasma concentration. After ruling out potential adverse drug reactions, the option of administering short‐term physiological doses of corticosteroids was discussed with the attending veterinarian. Most dogs experienced immediate benefit.

**Table 2:** Description of three general categories of clinical signs described in dogs with the MDR1 mutation, their respective response to corticosteroid treatment (if administered) and commonalities of results of diagnostic testing

General case description (all herding breed dogs)	MDR1 genotype	Subjective response to physiological doses of corticosteroid	Diagnostic testing commonalities
*Group 1* Dog(s) appear sicker than expected based on what physical exam, biochemical parameters and imaging would predict while failing to respond to generally accepted treatments	Usually MDR1 mutant/mutant	Some cases achieved dramatic improvement; some cases did not appear to achieve immediate benefit	Abdominal ultrasound: small adrenal glands Basal cortisol: low ACTH Stimulation: suboptimal but not diagnostic for hypoadrenocorticism
*Group 2* Vague clinical signs (lethargy, GI) with adrenal function testing not “low enough” to diagnose eunatraemic, eukalaemic hypoadrenocorticism	MDR1 mutant/normal MDR1 mutant/mutant	Improvement frequently noted but difficult to determine if concurrent treatments contributed to improvement	Abdominal ultrasound: small adrenal glands Basal cortisol: low ACTH stimulation: suboptimal but not diagnostic for hypoadrenocorticism
*Group 3* Dog(s) noticeably underperform athletically under hot/humid conditions compared to milder conditions	MDR1 mutant/normal MDR1 mutant/mutant	Corticosteroids not prescribed	Abdominal ultrasound: Small adrenal glands (one case)

The second group consists of dogs of known genotype (MDR1 mutant/mutant or MDR1 mutant/normal) that have displayed vague clinical signs often associated with eunatraemic eukalaemic hypoadrenocorticism (lethargy, inappetence, ± vomiting or diarrhoea), with ACTH stimulation test results suboptimal, but not diagnostic for hypoadrenocorticism (*i.e*. basal cortisol plasma concentrations slightly below laboratory reference ranges and post‐ACTH cortisol concentrations greater than the laboratory's cut‐off for diagnosing hypoadrenocorticism). In most instances, treatment with physiologic or supplemental doses of prednisone has improved clinical signs. In some dogs, appropriate tapering of prednisone resulted in recurrence of clinical signs, which then abated with reintroduction of prednisone. Pet owners and veterinarians reported prednisone doses ranging from 0.1 to 0.5 mg/kg that were successful in keeping clinical signs at bay in these dogs. In many instances, pet owners were hesitant to ever taper their dogs off prednisone, so it is presumed that treatment was continued indefinitely.

The third “group” consists of two dogs that compete in agility events located in multiple areas of the United States. Both dogs are Australian shepherds (one MDR1 mutant/mutant and one MDR1 mutant/normal), belonging to households with other Australian shepherds (MDR1 normal/normal) that compete in the same agility events. Owners reported that on multiple occasions, their P‐glycoprotein‐deficient dogs had noticeably poorer performances in hot/humid weather compared with their MDR1 normal/normal Australian shepherds, and that they were unable to attribute the poor performance to any other factor. Under conditions of milder weather, the P‐glycoprotein‐deficient Australian shepherds perform as well or better than the MDR1 normal/normal Australian shepherds. The author did not recommend corticosteroid supplementation in these instances since the dogs were otherwise healthy (but remains curious as to whether a physiological dose of prednisone would have improved performance).

## POTENTIAL THERAPEUTIC INTERVENTIONS

As has already been mentioned in this review, short‐term physiological doses of corticosteroids such as intravenous hydrocortisone for critically ill P‐glycoprotein‐deficient patients or oral prednisone (prednisolone for cats) for non‐critically ill P‐glycoprotein‐deficient patients have subjectively improved clinical signs. It is possible that some of the latter patients would benefit from longer term treatment, though this would have to be determined on an individual basis. With any pharmacological treatment, one must weigh the risks and benefits. However, if a patient is known to harbour the canine or feline MDR1 mutation and is experiencing clinical signs consistent with CIRCI or hypoadrenocorticism and has equivocal adrenal function test results, the potential benefits of short‐term treatment with physiological doses of corticosteroids likely outweigh potential risks.

When one appreciates P‐glycoprotein's role in the HPA axis, restricting hypothalamic cortisol concentrations, it is easy to see how animals with P‐glycoprotein deficiency would experience chronic suppression of the HPA axis. Indeed, adrenal function testing in both mice and dogs with P‐glycoprotein deficiency provides evidence of chronic HPA axis suppression in those animals. Similar studies should be performed in P‐glycoprotein‐deficient cats. Research in mice and anecdotal reports in dogs, and perhaps a cat, suggest that patients lacking P‐glycoprotein may benefit from corticosteroid supplementation in stressful situations. More research is necessary to determine if there are statistically significant benefits from corticosteroid supplementation in these patients, which corticosteroid(s) are indicated, and the optimal dose/dose interval. However, it seems the potential benefit of providing a physiological dose of corticosteroids outweighs the risk for P‐glycoprotein‐deficient patients in Groups 1 or 2 (*i.e*. acute illness or chronic eunatraemic, eukalaemic hypoadrenocorticism clinical signs) provided that appropriate diagnostic assessment and targeted treatments have been conducted. Treatment of Group 3 animals introduces ethical considerations. While evaluating athletic performance of MDR1 mutant/mutant dogs in heat/humidity *versus* milder conditions would provide interesting and potentially useful information, the ethics of corticosteroid supplementation to enhance performance would need to be addressed.

### Author contributions

**K. L. Mealey:** Conceptualization; investigation; writing – original draft; writing – review and editing; project administration; data curation.

### Conflict of interest

The author receives royalties from Washington State University for providing drug dosing recommendations for the apps MDR1Caddie® and Whispurr™.

## Data Availability

Data sharing is not applicable to this article as no datasets were generated or analysed during the current study.
